# RND3 Potentiates Proinflammatory Activation through NOTCH Signaling in Activated Macrophages

**DOI:** 10.1155/2024/2264799

**Published:** 2024-02-02

**Authors:** María José Romero de Ávila, Susana López-López, Aarón García-Blázquez, Almudena Ruiz-García, María Julia González-Gómez, María Luisa Nueda, Victoriano Baladrón, Ignacio Pérez-Roger, Enric Poch, Begoña Ballester-Lurbe, José Javier García-Ramírez, Eva M. Monsalve, María José M. Díaz-Guerra

**Affiliations:** ^1^Medical School, Biomedicine Institute (IB-UCLM)/Biomedicine Unit, University of Castilla-La Mancha/CSIC, C/Almansa 14, 02008, Albacete, Spain; ^2^Research Unit, University Hospital Complex of Albacete, C/Laurel s/n, 02008, Albacete, Spain; ^3^Biochemistry and Molecular Biology Branch, School of Pharmacy/CRIB/Biomedicine Unit, Department of Inorganic and Organic Chemistry and Biochemistry, University of Castilla-La Mancha/CSIC, Albacete, Spain; ^4^Department of Biomedical Sciences School of Health Sciences, University Cardenal Herrera-CEU, CEU Universities, 46115 Alfara del Patriarca, E-46115 Alfara del Patriarca, Valencia, Spain

## Abstract

Macrophage activation is a complex process with multiple control elements that ensures an adequate response to the aggressor pathogens and, on the other hand, avoids an excess of inflammatory activity that could cause tissue damage. In this study, we have identified RND3, a small GTP-binding protein, as a new element in the complex signaling process that leads to macrophage activation. We show that RND3 expression is transiently induced in macrophages activated through Toll receptors and potentiated by IFN-*γ*. We also demonstrate that RND3 increases NOTCH signaling in macrophages by favoring NOTCH1 expression and its nuclear activity; however, Rnd3 expression seems to be inhibited by NOTCH signaling, setting up a negative regulatory feedback loop. Moreover, increased RND3 protein levels seem to potentiate NF*κ*B and STAT1 transcriptional activity resulting in increased expression of proinflammatory genes, such as *Tnf*-*α*, *Irf-1*, or *Cxcl-10*. Altogether, our results indicate that RND3 seems to be a new regulatory element which could control the activation of macrophages, able to fine tune the inflammatory response through NOTCH.

## 1. Introduction

RND3, also known as RhoE, is a protein that belongs to the Rnd subclass of the small GTP-binding family of proteins [1, 2]. Unlike other GTPases, RND3 protein does not have the ability to hydrolyze GTP into GDP; therefore, its regulation is not based on the GTP/GDP cycle, but on modifications in the protein itself, such as phosphorylation or ubiquitination and changes in the expression of its gene [3, 4]. RND3 is expressed in various cell types, although its expression level and regulation vary from one cell type to another. The first identified biological function of RND3 was its role as a repressor of the RhoA/ROCK1 signaling pathway, which regulates actin dynamics [5]. RND3 participates in other cellular processes, such as regulation of cytoskeleton dynamics, proliferation, migration, and apoptosis [6–9]. However, more recently, numerous studies have linked RND3 to different types of cancer and to the development of the neuronal system [10–12]. Moreover, it has been documented that the expression of aberrant forms of RND3 may be related to the development of different diseases [1], including an important role in inflammatory diseases [13, 14].

Inflammation is an essential defense mechanism induced mainly by macrophages against infection and tissue damage. Inflammation is a process that allows the recruitment of cells and molecules to the infectious foci, triggering complex effector defense mechanisms to achieve the elimination of the threat [15]. When macrophages recognize pathogens through Toll-like receptors (TLRs), an inflammatory response is organized, including the synthesis and release of proinflammatory cytokines, such as tumor necrosis factor *α* (TNF*α*) or interleukin-6 (IL-6), among others [16]. Signaling through TLRs initiates a complex intracellular signaling cascade that leads to the activation of NF*κ*B, among others, increasing the expression of multiple target genes including proinflammatory cytokines [17]. IFN-*γ*, released by T-CD4^+^ Th1 and NK lymphocytes, strongly stimulates the activation of macrophages, synergizing with TLRs, and augmenting the production of inflammatory cytokines through the JAK/STAT1 pathway [18].

Different studies showed relevant roles of NOTCH receptors in the control of macrophage activation and in the induction of the inflammatory response [19–21]. NOTCH receptors can be cleaved at the internal side of the membrane, and the NOTCH-intracellular domains (NIC) are released and translocated to the nucleus; after translocation, they can modulate the expression of different genes. Notch intracellular domain then interacts with CBF-1/RBP-J DNA-binding proteins, and they form a transcriptional activator complex through the recruitment of coactivator proteins [22]. Different studies have shown that NOTCH1 and NOTCH3 promote macrophage activation by enhancing TLR and IFN-*γ* signaling [19, 23], whereas NOTCH4 acts as a negative regulator of macrophage activation interfering with IFN-*γ* signaling, by limiting STAT1-dependent transcription [24].

Although it has not been evaluated in macrophages, previous studies have shown a relationship between NOTCH receptors and RND3 in different cell types. For example, in squamous cell carcinoma [25] RND3 seems to mediate the translocation of the intracellular domain of NOTCH1 to the nucleus and its binding to the promoter of its target genes. However, other studies indicate that RND3 acts as a negative regulator of the NOTCH signaling, as it mediates the degradation of the intracellular domain of NOTCH1 by ubiquitination [9, 26, 27]. Our laboratory found *Rnd3* to be one of the genes differentially expressed in LPS and IFN-*γ* activated peritoneal macrophages modulated by NOTCH1 and NOTCH2 signaling. All these studies show that the role of RND3 in NOTCH activation and signaling is controversial and highly dependent on the cellular context.

In this study, we show that RND3 is transiently induced in macrophages activated through TLRs and IFN-*γ*, and that this process is modulated by NOTCH signaling. We also demonstrate that RND3 increases NOTCH signaling in macrophages and this process potentiates NF*κ*B and STAT1 activity resulting in the increased expression of proinflammatory genes as *Tnfα, Irf1*, or *Cxcl-10*. Therefore, RND3 seems to be a new regulatory element that could control macrophage activation able to fine tune the inflammatory response through NOTCH.

## 2. Results

### 2.1. RND3 Expression Is Induced in Macrophages Activated through TLRs and IFN-*γ* and Is Modulated by NOTCH Signaling

**Figure 1:**
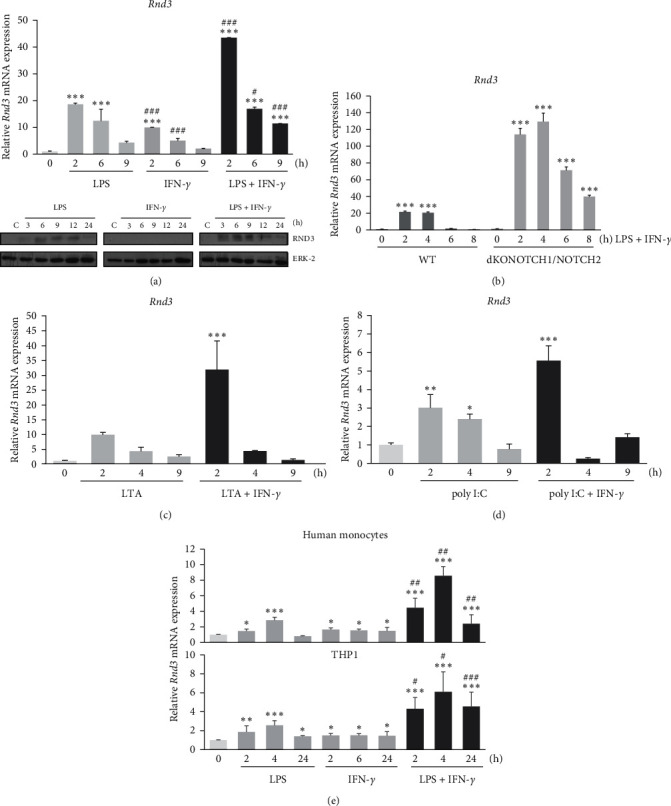
Analysis of the expression of *Rnd3* in macrophages activated through TLRs and IFN-*γ*. (a) qRT-PCR (upper panel) and Western blot (lower panel) analysis of *Rnd3* expression in peritoneal murine macrophages activated with LPS (100 ng/ml), IFN-*γ* (10 U/ml), or both for the indicated times. In Western blot analysis, ERK-2 expression was used as a loading reference. Means ± SD of three independent experiments are shown. One-way ANOVA/Bonferroni's post-tests was performed. Statistical significance was determined at the level of  ^*∗∗∗*^*p*  < 0.001, compared to control, untreated cells; ^#^*p*  < 0.05, ^###^*p*  < 0.001 with respect to the corresponding LPS-treated cells. (b) qPCR analysis of *Rnd3* expression in WT and Notch1/Notch2 knockout (dKONOTCH1/NOTCH2) peritoneal murine macrophages activated with LPS (100 ng/ml) and IFN-*γ* (10 U/ml) for different times. Means ± SD of three independent experiments are shown. One-way ANOVA analysis with Bonferroni's post-tests was performed. Statistical significance was determined at the level of  ^*∗∗∗*^*p*  < 0.001, with respect to corresponding untreated WT macrophages. qPCR analysis of *Rnd3* expression in peritoneal murine macrophages activated with (c) LTA (2 *μ*g/ml) or (d) Poly I:C (5 *μ*g/ml) in the presence or not of IFN-*γ* (10 U/ml) at different times. Means ± SD of at least three independent experiments are shown. One-way ANOVA analysis with Bonferroni's post-tests was performed. Statistical significance was determined at the level of  ^*∗*^*p*  < 0.05,  ^*∗∗*^*p*  < 0.01,  ^*∗∗∗*^*p*  < 0.001, compared to control untreated cells. (e) qPCR analysis of *Rnd3* expression in human monocytes or THP1 cells activated with LPS (100 ng/ml), IFN-*γ* (10 U/ml), or both for the indicated times. Means ± SD of three independent experiments are shown. One-way ANOVA analysis with Bonferroni's post-tests was performed. Statistical significance was determined at the level of  ^*∗*^*p*  < 0.05,  ^*∗∗∗*^*p*  < 0.001, compared to control untreated cells, ^#^*p*  < 0.05, ^##^*p*  < 0.01, ^###^*p*  < 0.001 with respect to the corresponding LPS-treated cells. All qPCR data are referred to unstimulated macrophages set to 1. qPCR was performed in triplicate with riboprotein P0 as the internal control.

To investigate the molecular mechanisms by which NOTCH receptors contribute to macrophage activation, we searched for new genes differentially expressed in LPS and IFN-*γ* activated peritoneal macrophages modulated by NOTCH signaling [28]. By using a set of Affymetrix microarrays, we identified *Rnd3*, among other genes, as a gene whose expression was activated by LPS and IFN-*γ* in macrophages and potentiated in the absence of NOTCH1/2 signaling. In order to confirm the microarray data, we used quantitative PCR, and as shown in Figure 1(a) (upper panel), we first observed that macrophages activated with LPS rapidly increased *Rnd3* mRNA levels, and the augmented *Rnd3* expression was enhanced by IFN-*γ*. Similar results were also observed by Western blot analysis, with a transient detection of RND3 in macrophages between 3 and 12 hr after activation (Figure 1(a), lower panel). We next aimed to confirm the effect of NOTCH signaling on *Rnd3* expression previously observed in the microarray analysis; as shown in Figure 1(b), *Rnd3* expression was strongly induced in activated macrophages lacking NOTCH1 and NOTCH2 receptors. To assess whether the increased expression of RND3 was a general process in TLR macrophage activation, we next used different Toll agonists to activate other TOLL receptors, Toll3 (poly I:C) and Toll2 (LTA). As Figures 1(c) and 1(d) show, in both cases *Rnd3* expression was rapidly and transiently induced, with a maximum of mRNA observed at 2 hr of activation; in both cases, the increased *Rnd3* expression was also potentiated by IFN-*γ*.

Lastly, we analyzed the expression of *Rnd3* in human monocytes and THP1 cells, and we could observe that its expression was also induced by LPS and potentiated by IFN-*γ* (Figure 1(e)). Taken together, our results indicate that the induction of *Rnd3* expression is a common event in the signaling process of macrophage activation, and that this process is potentiated in the absence of NOTCH1/2 receptors signaling.

### 2.2. RND3 Modulates NOTCH Signaling in Activated Macrophages by Increasing NOTCH1 Expression and Translocation to the Nucleus

**Figure 2:**
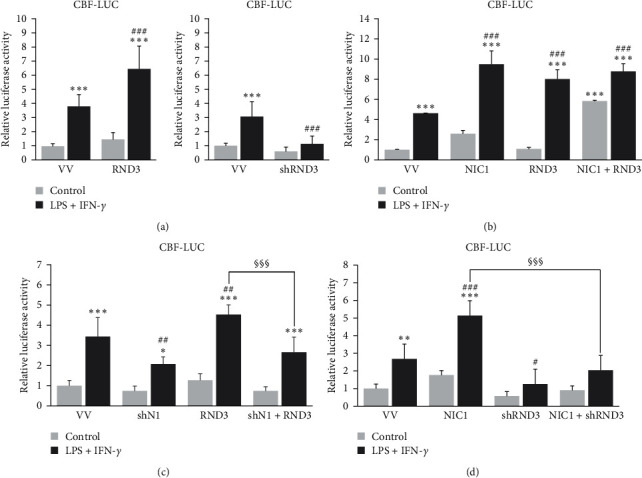
RND3 modulates NOTCH signaling in activated macrophages. (a) Analysis of NOTCH transcriptional activity in control and activated Raw 264.7 cells transiently transfected with a CBF luciferase reporter gene (CBF–LUC) in the presence of a RND3 expression vector (RND3) or its corresponding control vector (VV) (left panel) or *Rnd3*-specific shRNAs (shRND3) or its corresponding unspecific shRNA control vector (VV) (right panel). Means ± SD of four independent experiments are shown. One-way ANOVA analysis with Bonferroni's post-tests was performed. Statistical significance was determined at the level of  ^*∗∗∗*^*p*  < 0.001, compared to control untreated cells; ^###^*p*  < 0.001 compared to the corresponding control vector, treated cells. (b) Analysis of NOTCH transcriptional activity in control and activated Raw 264.7 cells transiently transfected with a CBF luciferase reporter gene (CBF–LUC) in the presence of a RND3 expression vector (RND3) or an intracellular active domain of NOTCH1 expression vector (NIC1) or both, and their corresponding controls vector (VV). Means ± SD of three independent experiments are shown. One-way ANOVA analysis with Bonferroni's post-tests was performed. Statistical significance was determined at the level of  ^*∗∗∗*^*p*  < 0.001, compared to control untreated cells; ^###^*p*  < 0.001 compared to the control vector, treated cells. (c) Analysis of NOTCH transcriptional activity in control and activated Raw 264.7 cells transiently transfected with a CBF luciferase reporter gene (CBF–LUC) in the presence of a RND3 expression vector (RND3) or Notch1-specific shRNA vector (shN1) or both, and their corresponding control vectors. Means ± SD of three independent experiments are shown. One-way ANOVA analysis with Bonferroni's post-tests was performed. Statistical significance was determined at the level of  ^*∗*^*p*  < 0.05,  ^*∗∗∗*^*p*  < 0.001, compared to control untreated cells; ^##^*p*  < 0.01, compared to the control vector, treated cells; ^§§§^*p*  < 0.001 compared to Rnd3-transfected, treated cells. (d) Analysis of NOTCH transcriptional activity in control and activated Raw 264.7 cells transiently transfected with a CBF luciferase reporter gene (CBF–LUC) in the presence of an active intracellular domain of NOTCH1 expression vector (NIC1) or *Rnd3*-specific shRNA (shRND3) or both, and their respective control vectors. Means ± SD of four independent experiments are shown. One-way ANOVA analysis with Bonferroni's post-tests was performed. Statistical significance was determined at the level of,  ^*∗∗*^*p*  < 0.01,  ^*∗∗∗*^*p*  < 0.001, compared to control untreated cells; ^#^*p*  < 0.05, ^###^*p*  < 0.001 compared to the control vector, treated cells; ^§§§^*p*  < 0.001 compared with cells transfected with intracellular active domain of NOTCH1 expression vector and control vector. Cells were stimulated with LPS (100 ng/ml) and IFN-*γ* (10 U/ml) for 24 hr before analysis, 1 day after transfection. pRLTK was used as an internal control vector for transfection and normalized luciferase/Renilla values are represented.

We and others have demonstrated that NOTCH1 is induced in activated macrophages and that signaling through this receptor potentiates the expression of proinflammatory and cytotoxic genes [20, 21]. Different authors have also shown that RND3 modulates NOTCH signaling, although opposing results are reported in different cell models [9, 25–27, 29]. So, we analyzed the role of RND3 in NOTCH signaling in Raw 264.7 cells transfected with a CBF-luciferase reporter gene, in the presence or not of a plasmid to overexpress RND3, and activated with LPS and IFN-*γ*. As Figure 2(a) shows, in the presence of high levels of RND3, the CBF-reporter gene activity was increased in all conditions. In the same line, diminished levels of RND3 by shRNAs caused a drastic inhibition of the CBF-reporter activity. These results clearly show that, in activated macrophages, RND3 potentiates NOTCH signaling.

As previous results obtained in squamous cells carcinoma showed that RND3 potentiates NOTCH signaling by favoring translocation of the intracellular domain of NOTCH1 to the nucleus [25], and considering that the expression of NOTCH1 is increased during macrophage activation [30], we tried to evaluate the role of NOTCH1 in RND3 potentiation of NOTCH signaling. As Figure 2(b) shows, both RND3 and the constitutively active intracellular domain of NOTCH1 (NIC) increased the activity of a CBF-reporter gene in activated macrophages. Moreover, when both proteins were overexpressed, CBF activity was potentiated in control macrophages, supporting the existence of a complementary role of each protein. However, in activated macrophages, the presence of both signals together did not enhance the effect of each individual signal, probably because the activation of macrophages already induced the expression of both (Figure 1; [21]). On the other hand, in the absence of NOTCH1, RND3 seems to be unable to increase NOTCH signaling (Figure 2(c)), whereas in the absence of RND3, transcriptional activity of the NOTCH target reporter gene by NIC was intensely diminished (Figure 2(d)). Altogether, our results indicate that the interaction of RND3 and NOTCH1 seems to be essential for the activation of NOTCH signaling in activated macrophages.

**Figure 3:**
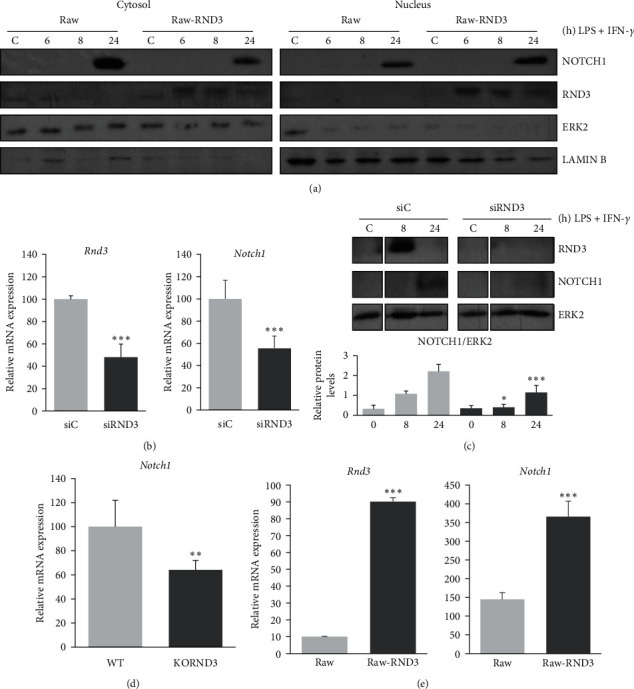
RND3 favors the translocation and expression of NOTCH1 in activated macrophages. (a) Western blot analysis of NOTCH1 expression in cytosol (left panel) and nucleus (right panel) in transiently transfected Raw 264.7 cells with control (Raw) or *Rnd3* expression vector (Raw-RND3) activated with LPS (100 ng/ml) and IFN-*γ* (10 U/ml). ERK-2 expression was used as a loading reference for cytosolic and total protein extracts, and LAMIN-B for nuclear extracts. (b) Analysis by qPCR of *Notch1*mRNA expression in activated peritoneal murine macrophages transfected with *Rnd3*-specific siRNA and treated with LPS (100 ng/ml) and IFN-*γ* (10 U/ml) for 3 hr (c) Analysis of NOTCH1 protein expression in murine macrophages treated as previously described. (d) qPCR analysis of *Notch1* in *Rnd3*^−/−^ bone marrow-derived macrophages (KORND3) and control macrophages (WT) activated 6 hr with LPS (100 ng/ml) and IFN-*γ* (10 U/ml). (e) qPCR analysis of *Notch1* in Raw 264.7 cells stably transfected with a control (Raw) or *Rnd3* expression vector (Raw-RND3) activated at 6 hr with LPS (100 ng/ml) and IFN-*γ* (10 U/ml). All qPCR data are referred to unstimulated macrophages set to 1. qPCR was performed in triplicate with riboprotein P0 as the internal control. Means ± SD of three independent experiments are shown. The Student unpaired *t*-test was used for statistical analyses between two groups at the level of  ^*∗*^*p*  < 0.05,  ^*∗∗*^*p*  < 0.01,  ^*∗∗∗*^*p*  < 0.001, compared to control untreated cells.

Previous results have shown that, in squamous cell carcinomas, RND3 was essential for the recruitment of intracellular domain of NOTCH1 to its target gene promoters by favoring NOTCH1 translocation to the nucleus [25]. We thus analyzed if NOTCH1 was enriched in the nuclear fraction of macrophages activated with LPS and IFN-*γ* in the presence of elevated levels of RND3. As Figure 3(a) shows, in the presence of high levels of RND3, the proportion of NOTCH1 in the nuclear fraction was increased compared with control cells. As NOTCH1 binds to its own promoter and induces its own expression [31], we next evaluated if RND3 would potentiate the expression of NOTCH1, by using specific siRNAs. As Figure 3(b) shows, the presence of *Rnd3* siRNAs diminished at least 50% the expression of *Rnd3* in murine macrophages, and in these same conditions, the expression of *Notch1* was also diminished. This effect could be clearly observed when relative protein levels were analyzed by Western blot (Figure 3(c)). In line with these results, expression of *Notch1* mRNA was lower in *Rnd3KO* bone marrow-derived macrophages (BMDM) than in control, WT cells (Figure 3(d)), and the overexpression of *Rnd3* in Raw 264.7 cells increased *Notch1* mRNA levels (Figure 3(e)). All these results show that RND3 increases NOTCH signaling in activated macrophages, at least in part, by increasing NOTCH1 expression and activity.

### 2.3. RND3 Modulates NF*κ*B Transcriptional Activity through NOTCH1

**Figure 4:**
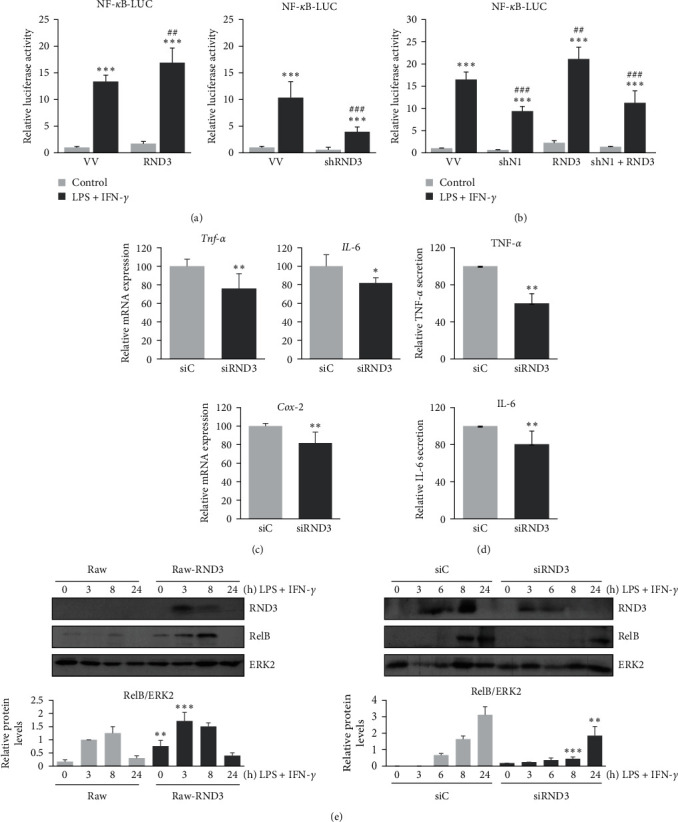
RND3 modulates NF*κ*B transcriptional activity. (a) Analysis of NF-*κ*B transcriptional activity in Raw 264.7 cells activated with LPS (100 ng/ml) and IFN-*γ* (10 U/ml) and transiently transfected with a NF-*κ*B luciferase reporter (NF*κ*B–LUC) and a *Rnd3* expression vector (RND3) or its corresponding control vector (VV) (left panel), or *Rnd3*-specific shRNA (shRND3) or unspecific shRNA control vector (VV) (right panel). Means ± SD of three independent experiments are shown. One-way ANOVA analysis with Bonferroni's post-tests was performed. Statistical significance was determined at the level of  ^*∗∗∗*^*p*  < 0.001, compared to control untreated cells; ^###^*p* < 0.001 compared to the corresponding control, treated cells. (b) Analysis of NF-*κ*B transcriptional activity in Raw 264.7 cells activated with LPS (100 ng/ml) and IFN-*γ* (10 U/ml) and transiently transfected with a NF-*κ*B luciferase reporter (NF*κ*B–LUC) and a Rnd3 expression vector (RND3), or *Notch1*-specific shRNA (shN1) or both. Means ± SD of three independent experiments are shown. One-way ANOVA analysis with Bonferroni's post-tests was performed. Statistical significance was determined at the level of  ^*∗∗∗*^*p*  < 0.001, compared to control untreated cells; ^##^*p*  < 0.01, ^###^*p*  < 0.001 compared to the corresponding control, treated cells. (c) qPCR analysis o*f Tnf*-*α, IL-6*, and *Cox-2* mRNA expression or (d) analysis of secreted TNF*α* and IL-6 by ELISA in peritoneal murine macrophages transfected with Rnd3-specific siRNA (siRND3) or control siRNA (siC) and activated for 6 hr with LPS (100 ng/ml) and IFN-*γ* (10 U/ml). All data are referred to stimulated macrophages treated with siControl RNA set to 100. qPCR was performed in triplicate with riboprotein P0 as the internal control. Means ± SD of three independent experiments are shown. Student unpaired *t*-test was used for statistical analyses between two groups at the level of  ^*∗∗*^*p*  < 0.01, compared to control, untreated cells. (e) Western blot analysis of *Rel-B* in Raw 264.7 cells transiently transfected with a control (Raw) or *Rnd3* expression vector (Raw-RND3) (left panel); and in peritoneal murine macrophages transfected with *Rnd3*-specific siRNA (siRND3) or control siRNA (siC) (right panel), activated with LPS (100 ng/ml) and IFN-*γ* (10 U/ml). ERK-2 expression was used as a loading reference. A representative image of at least three experiments is shown. Means ± SD of three independent experiments are shown. One-way ANOVA analysis with Bonferroni's post-tests was performed. Statistical significance was determined at the level of  ^*∗*^*p* < 0.05,  ^*∗∗*^*p*  < 0.01,  ^*∗∗∗*^*p*  < 0.001 compared to the corresponding empty vector, untreated cells.

NOTCH1 increases NF*κ*B activity in different cell models, including TLR activated macrophages [21, 30, 32]; besides, we have demonstrated that RND3 is induced in response to TLR activation and potentiates NOTCH signaling. Thus, we evaluated if RND3 could affect NF*κ*B transcriptional activity after macrophage activation by using an NF*κ*B reporter gene. As Figure 4(a) shows, RND3 increased the NF*κ*B reporter activity induced by the treatment with LPS and IFN-*γ*, whereas diminished expression of RND3 by shRNAs clearly lowered NF*κ*B reporter activity. Moreover, transfection with a specific *Notch1* shRNA produced no potentiation of RND3 on NF*κ*B reporter gene activity (Figure 4(b)). So, RND3 induced in response to TLR signaling would potentiate NF*κ*B activation, a key transcription factor in the expression program of proinflammatory macrophages, at least in part through its effect on the expression of NOTCH1. In agreement with these results, the expression of *Tnf-α*, *Cox-2*, and *Il-6* was diminished in macrophages transfected with *Rnd3* siRNA and treated with LPS and IFN-*γ* (Figures 4(c) and 4(d)).

To evaluate NF*κ*B activation, we also analyzed the expression of RELB, a NF*κ*B subunit which is induced by the own NF*κ*B signaling pathway [30, 33], in the presence of elevated or reduced levels of RND3, and we observed that higher expression of RND3 increased *RelB* expression, while lowering RND3 expression diminished the relative expression of *RelB* (Figure 4(e)). As the expression of *RelB* depends on NF*κ*B activity, RND3 could favor a positive feedback loop that would increase the expression of NF*κ*B-dependent genes.

### 2.4. RND3 Modulates IFN-*γ* Signaling in Macrophages

**Figure 5:**
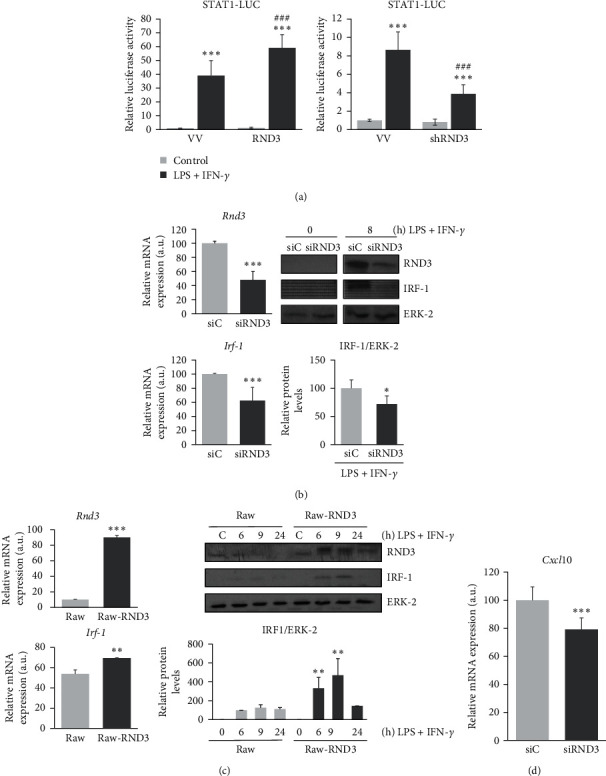
RND3 modulates STAT1 transcriptional activity in activated macrophages. (a) Analysis of STAT1 transcriptional activity in Raw 264.7 cells activated with LPS (100 ng/ml) and IFN-*γ* (10 U/ml) and transiently transfected with a STAT1 luciferase reporter (STAT1–LUC) and a *Rnd3* expression vector (RND3) or its corresponding control vector (VV) (left panel), or *Rnd3*-specific shRNAs (shRND3) or unspecific control shRNA vector (VV) (right panel). Means ± SD of three independent experiments are shown. One-way ANOVA analysis with Bonferroni's post-tests was performed. Statistical significance was determined at the level of  ^*∗∗∗*^*p*  < 0.001, compared to control, untreated cells; ^###^*p*  < 0.001 compared to the corresponding control, treated cells. (b) qPCR analysis of *Rnd3* and *Irf-1* mRNA expression (left panel), or western blot analysis of RND3 or IRF1 (right panel) in peritoneal murine macrophages transfected with *Rnd3*-specific siRNA (siRND3) or control siRNA (siC) and activated for 6 hr with LPS (100 ng/ml) and IFN-*γ* (10 U/ml). qPCR data are referred to control stimulated macrophages set to 100. qPCR was performed in triplicate with riboprotein P0 as the internal control. Means ± SD of three independent experiments are shown. Student unpaired *t*-test was used for statistical analyses between two groups at the level of  ^*∗*^*p*  < 0.05,  ^*∗∗∗*^*p*  < 0.001, compared to control untreated cells. (c) qPCR analysis of *Rnd3* and *Irf-1* mRNA expression (left panel), or Western blot analysis of RND3 or IRF1 (right panel) in Raw 264.7 cells transiently transfected with a control (Raw) or RND3 expression vector (Raw-RND3) activated with LPS (100 ng/ml) and IFN-*γ* (10 U/ml). ERK-2 expression was used as a loading reference. A representative image of at least three independent experiments is shown in Western blot analysis. One-way ANOVA analysis with Bonferroni's post-tests was performed. Statistical significance was determined at the level of  ^*∗∗*^*p*  < 0.001,  ^*∗∗∗*^*p*  < 0.001, compared to control untreated cells. (d) qPCR analysis of *Cxcl10* mRNA expression in peritoneal murine macrophages transfected with *Rnd3*-specific siRNA (siRND3) or control siRNA (siC) and activated for 6 hr with LPS (100 ng/ml) and IFN-*γ* (10 U/ml). qPCR data are referred to control stimulated macrophages set to 100. qPCR was performed in triplicate with riboprotein P0 as the internal control. Means ± SD of three independent experiments are shown. Student unpaired *t*-test was used for statistical analyses between two groups at the level of  ^*∗∗∗*^*p*  < 0.001, compared to control untreated cells.

Since signaling through NOTCH1 potentiates IFN-*γ* signaling in macrophages [20], we also analyzed the effect of RND3 on the IFN-*γ* signal transduction by using a reporter gene containing STAT1 binding sites. As Figure 5(a) shows, activation of macrophages with LPS and IFN-*γ* intensely induced the reporter gene activity. This effect was potentiated in cells with an elevated expression of RND3, whereas in macrophages with reduced levels of this protein, the effect was diminished. We next analyzed the expression of IRF1, a STAT1 target gene, in activated macrophages, in the presence of control or *Rnd3* specific siRNAs. As Figure 5(b) shows, macrophages with reduced RND3 mRNA levels showed lower relative expression of IRF1 mRNA and protein. On the contrary, in the presence of higher levels of RND3, the induction of IRF1 expression was potentiated (Figure 5(c)).

We also evaluated the levels of some cytokines whose expression depends on IFN-*γ*/STAT1/IRF-1 signaling, as CXCL10, and we observed a diminished expression of the protein CXCL10 when RND3 levels were lowered (Figure 5(d)). All these results show that RND3 potentiates IFN-*γ* signaling in activated macrophages.

## 3. Discussion

**Figure 6:**
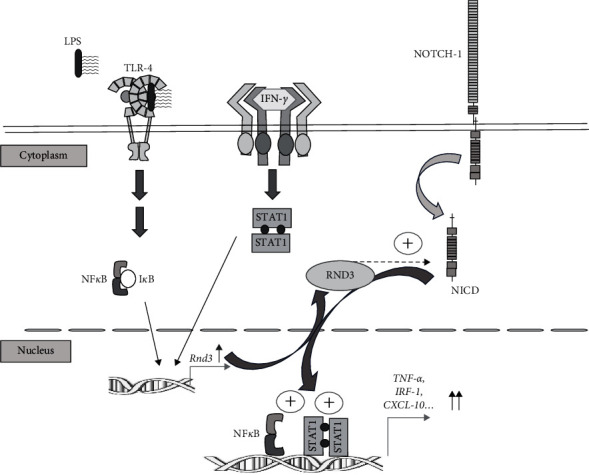
Schematic representation of RND3 induction and interaction with TLR4 and IFN*γ* receptor signaling in activated proinflammatory macrophages. RND3 is transiently induced in macrophages activated through TLR-4 and IFN-*γ*. Afterward RND3 potentiates NOTCH1 activation in macrophages and this process enhances the NF*κ*B and STAT1 transcriptional activity induced by TLR-4 and IFN-*γ* signaling pathway, respectively, resulting in the increased expression of STAT1 and NF-*κ*B proinflammatory genes target genes, such as *Tnf*-*α, Irf-1*, or *Cxcl-10* among others.

To carry out their functions, macrophages activate powerful effector mechanisms to deal with infections by multiple pathogens. Macrophage activation is a complex process with multiple control elements that ensure an adequate response to the aggressor element and, on the other hand, avoid an excess of activity that could cause tissue damage. In this study, we identified RND3 as a new element in the complex signaling process that leads to macrophage activation. We show that RND3 is transiently induced in macrophages activated through TLRs and IFN-*γ*, and that this process is modulated by NOTCH signaling. We demonstrate that RND3 increases NOTCH signaling in macrophages and that this process potentiates NF*κ*B and STAT1 transcriptional activity, resulting in the increased expression of proinflammatory genes such as *Tnf*-*α*, *Irf-1*, or *Cxcl-10* (Figure 6). Therefore, RND3 seems to be a potential new regulatory element in the control of macrophage activation able to fine tune the inflammatory response through notch.

First reports showed that RND3 induced actin reorganization in macrophages, favoring the formation of extensions resembling filopodia and pseudopodia that are essential for a proper migration and phagocytosis [5]. Subsequent studies showed that RND3 binds to the kinase ROCK1 and inhibits its activation [8], and that ROCK1 phosphorylates RND3 increasing its stability [34]. In macrophages, ROCK1 seems to be implicated in negative regulation of macrophage and neutrophil migration [35]. Although interaction of RND3 with ROCK kinases, as a repressor of RhoA/ROCK1 signaling pathway, is the first identified functional role of RND3, other interactions of RND3 with other signaling pathways have been described, including that of NOTCH receptors. In an early report related to the differentiation of *Xenopus* embryos, a correlation of NOTCH signaling to *Rnd3* expression was suggested [36].

Our results show that RND3 is transiently induced in macrophages activated by TLRs and IFN-*γ*, and that NOTCH1 and NOTCH2 signaling seems to limit this induction, as *Rnd3* expression is higher in macrophages that lack those receptors. Moreover, RND3 potentiates transcriptional activity of NOTCH in macrophages, increasing the amount of NOTCH1 intracellular domain in the nuclear fraction, and increasing NOTCH1 expression. Thus, in macrophages, RND3 increases *Notch1* expression and NOTCH1 signaling limits *Rnd3* expression in a clear negative regulatory feedback. Different reports have described interactions of RND3 with the NOTCH signaling pathway, although contradictory results have been found in different cellular models. Our results agree with those observed in squamous cell carcinomas, where RND3 depletion suppresses NOTCH1-mediated signaling. In this model, RND3 seems to mediate the nuclear translocation of NICD by favoring the binding of NICD to importin-b1 and thus ensuring the proper translocation of NICD to the nucleus. However, in that model, NOTCH signaling increases *Rnd3* expression creating a positive feedback loop [25], instead of the negative feedback loop that we found. Nevertheless, other studies made in glioblastoma cells showed that RND3 is a negative regulator of NOTCH signaling, as RND3 mediates NICD1 protein degradation through promoting its ubiquitination [26]. Moreover, downregulation of RND3 in glioblastoma patients promotes tumorigenesis through augmentation of NOTCH complex transcriptional activity [27]. Recent results obtained in fibroblast show also that NOTCH and TGF-*β* signaling were significantly suppressed upon Rnd3 overexpression [29]. All these results show that the function of NOTCH signaling and its relationship with RND3 is cell-context dependent, and that further investigation needs to be done to completely establish their complex interaction.

Another important conclusion of our results is that RND3 increases NF*κ*B transcriptional activity in activated macrophages, favoring the expression of some proinflammatory cytokines or enzymes, such as TNF-*α* or COX-2. We and others have previously shown that NOTCH1 and NOTCH3 potentiate NF*κ*B activation [19–21], so we expected to observe that RND3 increases NF*κ*B transcriptional activity, probably through an increased in NOTCH1 expression. Our results confirm this hypothesis, since RND3 could not increase the NF*κ*B signal in macrophages with decreases expression of NOTCH1. Similar results were shown by a previous report that described that ethanol induced the expression of RND3 in astrocytes, and that this protein stimulated the IRAK/ERK/NF*κ*B pathway and COX2 expression [14]. Nevertheless, different and controversial results are found in different cell types. Hearts of Rnd3^−/−^ mice show a significant upregulation of proinflammatory factors, including cytokines and chemokines, as detected in a mRNA microarray. In *Rnd3*-deficient cardiomyocytes, an increase of nuclear p65 and p50 was detected, as well as a blockade of the NF*κ*B translocation to the nucleus due to a direct interaction between RND3 and p65 and p50 [13]. These results indicate that, in cardiomyocytes, RND3 acts as a suppressor of NF*κ*B activity. In line with these results, in human glioblastoma multiforme cells, overexpression of RND3 reduced p65 activity. In this case, RND3 seems to bind to p65 favoring its ubiquitination and degradation and diminishing NF*κ*B signaling [37]. NF*κ*B p65 was upregulated in the brains of Rnd3^−/−^ mice compared with wild-type mice, and an interaction of p65 with RND3 was detected in PC12 cells, which showed a decrease of NF*κ*B signaling in the presence of RND3 [6]. The disparity of results observed in the interaction of RND3 and NF*κ*B might be explained, at least in part, because in most of the cell models described there is a basal level of expression of RND3, and an increase of p65 activity is observed when *Rnd3* expression is reduced. However, our results are observed in a context in which there is no basal expression of RND3, and there is a transitory and coincident *Rnd3* induction and NF*κ*B activation as a result of Toll receptor signaling. In this sense, it has been described that in macrophages, the inhibition of ROCK1/2 enhanced the release of TNF-*α* in response to the stimulation of TLR4. ROCK1/2 inhibition enhanced phosphorylation of the TLR downstream signaling molecules, p38, ERK1/2, and NF*κ*B [38]. As previously described, RND3 binds to the kinase domain of ROCK1 and inhibits the catalytic activity of ROCK1 [8]. That could explain the effect of RND3 on NFkB activity in the context of TLR activated macrophages.

We have observed that RND3 expression is induced by TLR signaling and potentiated by IFN-*γ*, although more studies are needed to identify the transcriptional factors implicated in *Rnd3* expression. It is known that hypoxia-inducible factor-1*α* (HIF-1*α*) directly binds to the Rnd3 gene promoter and drives its expression in gastric cancer cells [39]. It could be possible that HIF-1*α* acted as a mediator of RND3 expression in TLR activated macrophages, as TLR signaling induces HIF-1 expression via NF*κ*B [40]. Indeed, it has been described that RND3 physically interacts with and stabilized HIF-1*α*, and in this way promotes the expression of genes such as VEGFA [41].

Different studies have identified RND3 as a protective factor for mitochondrial function; indeed, the absence of RND3 activity has been related with altered mitochondria oxidative metabolism, favoring dependence of cells on glycolysis to obtain energy [42, 43]. In other cellular models, the absence of RND3 has been related with an increase of ROS generation and mitochondria dysfunction; moreover, a physical interaction between PPAR*γ* and RND3 has been described to potentiate mitochondrial function [44]. Further studies are needed to evaluate if TLR/IFN-*γ* induced expression of Rnd3 has implications in metabolic adaptation of activated macrophages.

The expression of other Rho GTPases as RhoA and RhoB are also modulated by TLR activation. In macrophages, knockdown of *RhoB* expression markedly decreased TLR induced activation of mitogen activated protein kinases and NF*κ*B, and the production of tumor necrosis factor-*α* (TNF-*α*), *IL-6*, and *IL-1β* [45]. Moreover, mutations on RhoA that favored immunosuppressive ambience in tumor microenvironment have been recently described [46].

In this study, we have identified RND3 as a new gene transiently expressed in macrophages activated through TLRs and IFN-*γ*, able to modulate both macrophage activation and the inflammatory response through NOTCH. Our work supports that GTPases play a relevant role in macrophage activation and points to these proteins as new regulatory elements able to fine tune the inflammatory response.

## 4. Materials and Methods

### 4.1. Mice

C57BL/6 mice were purchased from Jackson Laboratories (Farmington, CT, USA). Rnd3 deficient mice (Rnd3^−/−^) were kindly provided by Dr. Enric Poch (Faculty of Health Sciences, University CEU Cardenal Herrera, Valencia, España) [47]. All procedures conducted with mice were approved by the Committee for Ethics in Animal Care of the University of Castilla-La Mancha and followed the European and Spanish regulations.

### 4.2. Cells and Reagents

Peritoneal macrophages were isolated as previously described [30] from 2-month-old male mice, 4 days after i.p. injection of 2 ml sterile thioglycolate broth (3% w/v in water, Gibco). Elicited macrophages were seeded at a confluence of 1 × 10^5^ cells/cm^2^ in complete DMEM medium (supplemented with 2 mM L-glutamine, 10% FBS-fetal bovine serum-, and 1% penicillin-streptomycin, all from Lonza) and incubated in complete DMEM medium overnight, with 2% FBS, before the addition of either 100 ng/ml LPS (Salmonella typhimurium, Sigma–Aldrich), 200 ng/ml of poly I : C (Amersham Bioscience), 5 *μ*g/ml of lipoteichoic acid (LTA) from *Staphylococcus aureus* (InvivoGen) and/or 10 U/ml of interferon-*γ* (mouse mIFN-*γ*, Roche or human hIFN-*γ*, Roche). Activation was verified by the Griess test.

As Rnd3 KO mice life expectancy is about 1 month [47] and, under these conditions, the Animal Ethics Committee did not allow the use of intraperitoneal injection of thioglycolate to cause the extravasation of macrophages to the peritoneum, we isolated BMDM from a one-month-old WT and *Rnd3 KO mice* as previously described [48].

Raw 264.7 cells were acquired from ATCC (ATCC No.TIB-71) and subcultured at 6–8 × 10^4^ cells/cm^2^ in DMEM medium (Lonza) with 10% FBS, 1% penicillin–streptomycin and 4 mM L-glutamine and incubated overnight in complete DMEM supplemented with 5% FBS, preceding activation with cytokines. Cell activation was evaluated in each experiment by checking the production of nitrites (Griess reaction) after proinflammatory stimuli.

THP-1 cells were acquired from ATCC (ATCC no. TIB-202) and cultured in RPMI-1640 medium (Lonza) with 10% FBS, 1% penicillin–streptomycin and 4 mM L-glutamine, 0.05 mM 2-mercaptoethanol, and 10% FBS. After cells were differentiated during 24 hr into macrophages in FBS free RPMI-1640 with 5 ng/ml PMA, cells were cultivated for another 24 hr in serum-free medium, prior to activation. All cell lines were regularly checked for the absence of mycoplasma infection.

Human monocytes were isolated from healthy donors by centrifugation on Ficoll-PaqueTM PLUS (Amersham Biosciences), following the standard procedure [49] and cultured in complete DMEM medium with or without 100 ng/ml LPS for 24 hr. Human samples were processed under the European Union and Spanish regulations.

### 4.3. Cell Transfections

For transient transfections, 2.5 × 10^5^ Raw 264.7 cells/well were seeded in triplicate on 24-well plates and transfected with Lipofectamine 2000 (Invitrogen) on the following day, according to the manufacturer's recommendations, by using OPTI-MEM medium (Gibco) without supplements and 1.25 *µ*g/well of total EndoFree plasmid DNA. The reporter plasmids pNF-*κ*B-luc, pCBF-luc, and pSTAT1-luc, were used to detect NF-*κ*B, STAT1, and NOTCH-dependent transcription activities, respectively, as previously described [20, 30]. pRLTK Renilla-expressing vector (Promega) was used for the control of transfection efficiency. Sh-Control, Sh-Notch1 and Sh-Rnd3 (eBioscience), pLNCX2 (empty vector, BD Bioscience) and/or pLNCX2-NIC1 (intracellular domain Notch1 expression vector), pCMV6 (empty vector, Origene), and pCMV6-Rnd3 (eBioscience) were used together with the reporters. Cells were stimulated for 24 hr after being transfected. Luciferase and Renilla enzymatic activities were measured with Dual Luciferase Reporter Assay System (Promega) in “Orion II Microplate Luminometer” (Berthold) following the manufacturer's recommendations.

### 4.4. siRNA Silencing

Peritoneal macrophages were seeded on six-well plates to a cell density of 1.5 × 10^6^ cells per well. The following day, macrophages were transfected with 50 nM siRNAs anti-Rnd3 (Dharmacon) or the corresponding scrambled siRNA control (Dharmacon), using a Lipofectamine^TM^ RNAiMAX reagent (Invitrogen), following the manufacturer's recommendations. Cells were stimulated 72 hr after transfection. Sequences of the mixture of siRNAs are shown in Table 1.

### 4.5. Protein Extracts and Western Blot Analysis

Cells were washed twice with ice-cold PBS, they were scraped off the dishes and collected by centrifugation. All cell pellets were resuspended in lysis buffer (RIPA: 25 mM Hepes, pH 7.5; 1.5 mM MgCl_2_; 0.2 mM EDTA; 1% Triton X-100; 20 mM *β*-glycerophosphate; 0.3 M NaCl; 0.1% SDS; 0.5% deoxycholic acid) supplemented protease and phosphatase inhibitors (Sigma–Aldrich), homogenized for 30 min and centrifuged at 8,000x *g* for 15 min at 4°C. Protein concentrations were measured by the bicinchoninic acid method (Bio-Rad, Hercules, CA, USA).

Denatured protein extracts (40–80 *µ*g) were electrophoresed by SDS-PAGE in 10% polyacrylamide gels, then transferred to PVDF membranes (Sigma–Aldrich), and processed according to the recommendations of the antibody suppliers. Luminescence was detected with ECL (Santa Cruz, Biotechnology Dallas, TX, USA). ERK2 and LAMIN B expression were used as loading controls for cytosolic and nuclear extracts, respectively. If possible, we used different antibodies on the same blots, unless the expected molecular weight of the proteins coincided, in which case, we removed the bound antibody by incubation with a stripping buffer (100 mM *β*-mercaptoethanol, 2% sodium dodecyl sulfate, 62.5 mM Tris HCl pH 6.7) at 55°C for 30 min, prior to the second blotting.

Anti-IRF1 (8478), anti-RelB (4922), and anti-LAMIN B (12586) were purchased from cell signaling. Anti-RND3 was purchased for Merck Millipore. NOTCH1 (Ab52627) was purchased from Abcam. Anti-ERK-2 (sc-154) was purchased from Santa Cruz Biotechnology.

Western blots were scanned and analyzed using Quantity One software (BioRad). Each protein band was measured and normalized to the corresponding ERK-loading control band. The original scans are shown in Figure S1.

### 4.6. RNA and cDNA Purification

Total RNA was obtained by using the *NZY Total RNA Isolation kit* and DNase (NZYTech, Lisboa, Portugal), according to the manufacturer's instructions, and quantified in a NanoDrop One/One^c^ (Thermo Fisher Scientific, Waltham, MA, USA). cDNA was synthesized starting with 1 *µ*g of total RNA, using the RevertAidH Minus First Strand cDNA Synthesis (Thermo Fisher Scientific) and following manufacturer's recommendations.

### 4.7. Quantitative RT-PCR

Gene expression analysis by quantitative RT-PCR (qRT-PCR) was performed in triplicates according to the Fast SYBR® Green Protocol with the StepOne real-time PCR detection system (Applied Biosystems). Specific oligonucleotides were designed with PrimerQuest SM (Integrated DNA Technologies, Inc., Coralville IA, USA) and are indicated in Table 2. The mRNA levels of the human GAPDH or mouse riboprotein P0 [50] were used as internal controls [28].

### 4.8. Statistical Analysis

Values represent mean ± SEM. All statistical analyses were performed using IBM SPSS Statistics (IBM Corp). Statistical comparisons between two groups were performed using the Student's unpaired *t*-test, and for more than two groups, one-way ANOVA, with Bonferroni's post-tests, was performed.

## Figures and Tables

**Table 1:** Sequences of siRNAs.

siControl	siRnd3
UAAGGCUAUGAAGAGAUAC	GAACGUGAAAUGCAAGAUA
AUGUAUUGGCCUGUAUUAG	AGACUCCUGUGUCAUAUGA
AUGAACGUGAAUUGCUCAA	AAUCGACACACAAAGAAUA
UGGUUUACAUGUCGACUAA	CGGAGCAGCCACUUACAUA

**Table 2:** Sequence of specific oligonucleotides used in qRT-PCR.

Gene	Forward primer	Reverse primer
m*P0*	5′-GAATCGCTCCTGCAGCAAAG-3′	5′-CCAGGGTCTCATCCGCATT-3′
h*GADPH*	5′-AACCCTTGGCATTGTGGAAGG-3′	5′-GGATGCAGGGATGATGTTCT-3′
h*RND3*	5′-AGAAGAGCCAGCCAGAAATTAT-3′	5′-CCCACAGGCTCAACTCAACTCTATTC-3′
m*Notch1*	5′-TGTCTATGCCAGGCTAATGAAG-3′	5′-AGGGTGAGCAGGAACATGAG-3′
m*Rnd3*	5′-GTCCCTACGGTGTTTGAGAATTA-3′	5′-GACGGACGTTGTCATAGTAAGG-3′
mTnf-*α*	5′-CTATGTCTCAGCCTCTTCTC-3′	5′-CATTTGGGAACTTCTCATCC-3′
m*Cox-2*	5′-TGCCTCCCACT CAGACTAGA-3′	5′-TGCCTCCCACTCCAGACTAGA-3′
m*Irf-1*	5′-GAATCGCTCCTGCAGCAAAG-3′	5′-GAATCGCTCCTGCAGCAAAG-3′
m*Cxcl10*	5′-TCAGGCTCGTCAGTTCTAAGT-3′	5′-CCTTGGGAAGATGGTGGTTAAG-3′

## Data Availability

All the data generated during this project have been intended to unveil our scientific objectives, and data have been managed according to fair data policies. Digital data obtained in the laboratory have been stored in a shared folder located in the UCLM servers. Data are routinely accessible to personnel of our research group, with occasional access of other interested researchers. We have not produced any functional genomics data. Data meet the ethical standards; we have not used personal information and have followed the Spanish and European Union legislation.

## Supplementary Materials

Supplementary Materials Figure S1: full scans of each western blot, including the protein ladder information and the corresponding control, are shown for every figure.
