# Supplementary material for: RND3 Potentiates Proinflammatory Activation through NOTCH Signaling in Activated Macrophages
Source: J Immunol Res. 2024 Feb 2;2024:2264799. doi: 10.1155/2024/2264799 (PMC10857877; doi:10.1155/2024/2264799)
Supplement: Supplementary Materials — Figure S1: full scans of each western blot, including the protein ladder information and the corresponding control, are shown for every figure. [file 2264799.f1.pdf]

Supplementary figures

Full scans of each western blot, including the protein Ladder information and the corresponding control, are shown for every figure.

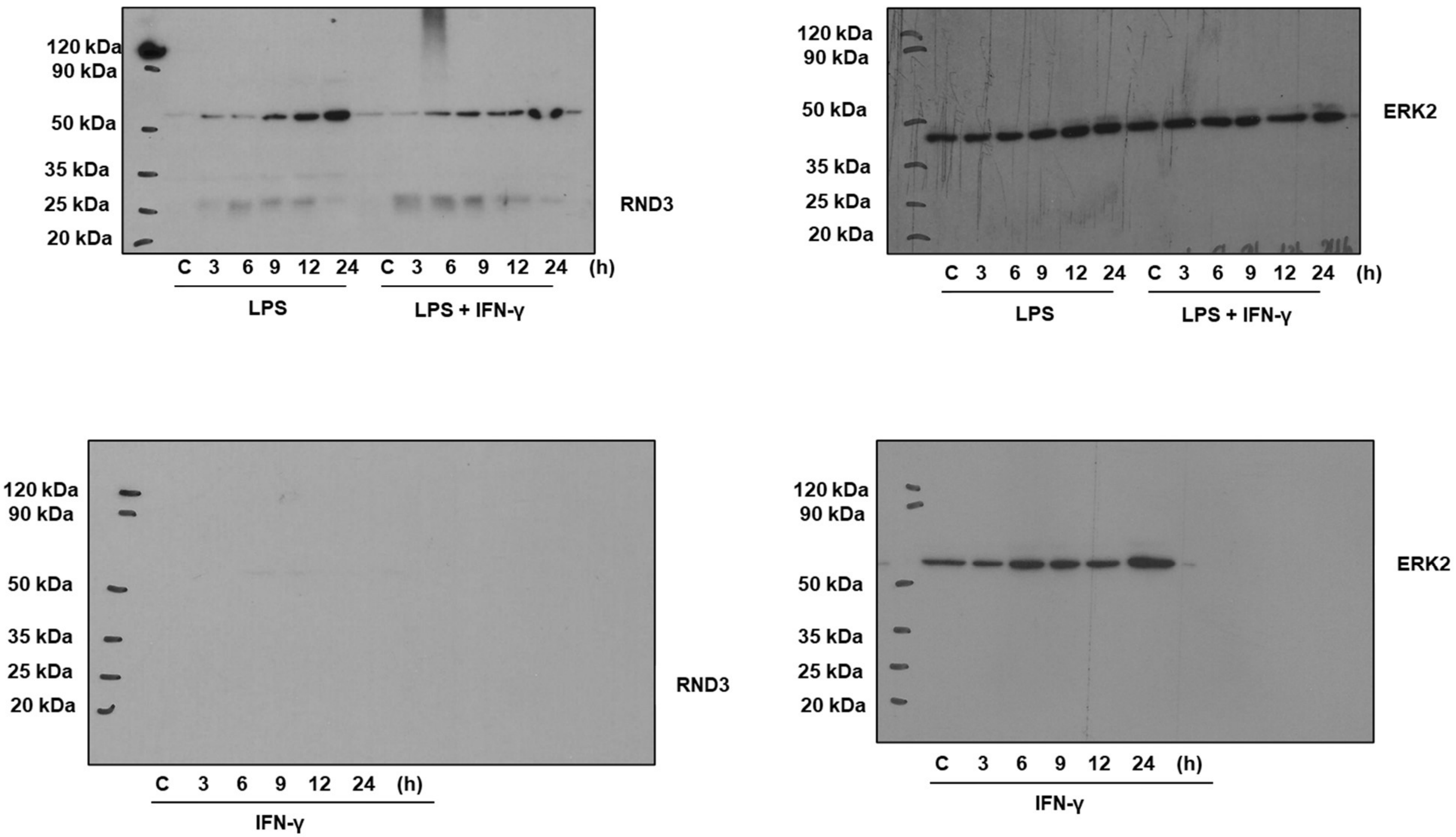

120 kDa
90 kDa
50 kDa
35 kDa
25 kDa
20 kDa
RND3
C 3 6 9 12 24 C 3 6 9 12 24 (h)
LPS
LPS + IFN-γ
120 kDa
90 kDa
50 kDa
35 kDa
25 kDa
20 kDa
ERK2
C 3 6 9 12 24 C 3 6 9 12 24 (h)
LPS
LPS + IFN-γ
120 kDa
90 kDa
50 kDa
35 kDa
25 kDa
20 kDa
RND3
C 3 6 9 12 24 (h)
IFN-γ
120 kDa
90 kDa
50 kDa
35 kDa
25 kDa
20 kDa
ERK2
C 3 6 9 12 24 (h)
IFN-γ

Figure 1A

Figure 3A

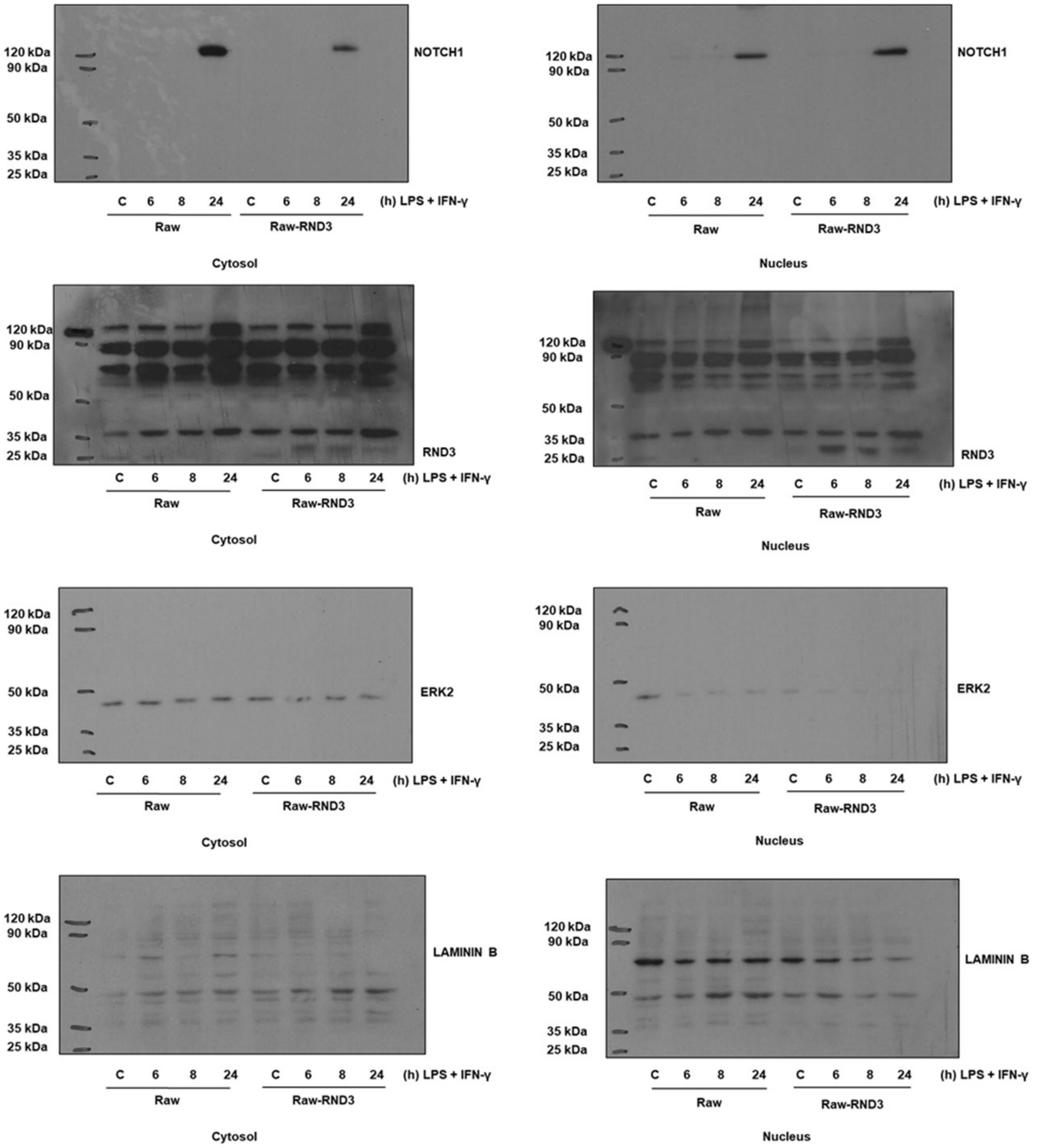
120 kDa
90 kDa
50 kDa
35 kDa
25 kDa
NOTCH1
C 6 8 24 C 6 8 24 (h) LPS + IFN-γ
Raw
Raw-RND3
Cytosol
120 kDa
90 kDa
50 kDa
35 kDa
25 kDa
NOTCH1
C 6 8 24 C 6 8 24 (h) LPS + IFN-γ
Raw
Raw-RND3
Nucleus
120 kDa
90 kDa
50 kDa
35 kDa
25 kDa
RND3
C 6 8 24 C 6 8 24 (h) LPS + IFN-γ
Raw
Raw-RND3
Cytosol
120 kDa
90 kDa
50 kDa
35 kDa
25 kDa
RND3
C 6 8 24 C 6 8 24 (h) LPS + IFN-γ
Raw
Raw-RND3
Nucleus
120 kDa
90 kDa
50 kDa
35 kDa
25 kDa
ERK2
C 6 8 24 C 6 8 24 (h) LPS + IFN-γ
Raw
Raw-RND3
Cytosol
120 kDa
90 kDa
50 kDa
35 kDa
25 kDa
ERK2
C 6 8 24 C 6 8 24 (h) LPS + IFN-γ
Raw
Raw-RND3
Nucleus
120 kDa
90 kDa
50 kDa
35 kDa
25 kDa
LAMININ B
C 6 8 24 C 6 8 24 (h) LPS + IFN-γ
Raw
Raw-RND3
Cytosol
120 kDa
90 kDa
50 kDa
35 kDa
25 kDa
LAMININ B
C 6 8 24 C 6 8 24 (h) LPS + IFN-γ
Raw
Raw-RND3
Nucleus

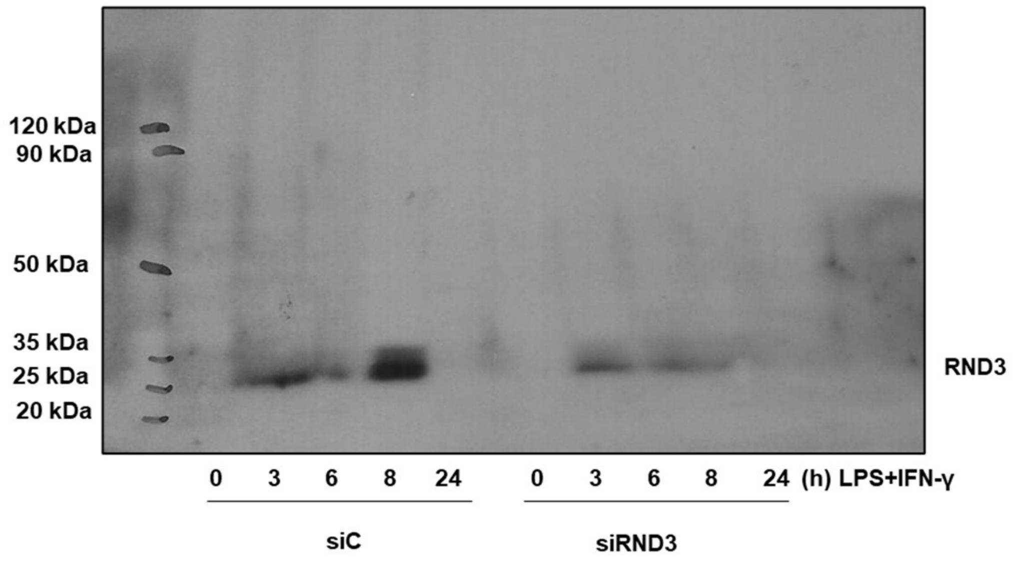
120 kDa
90 kDa
50 kDa
35 kDa
25 kDa
20 kDa
RND3
0 3 6 8 24 0 3 6 8 24 (h) LPS+IFN-γ
siC
siRND3

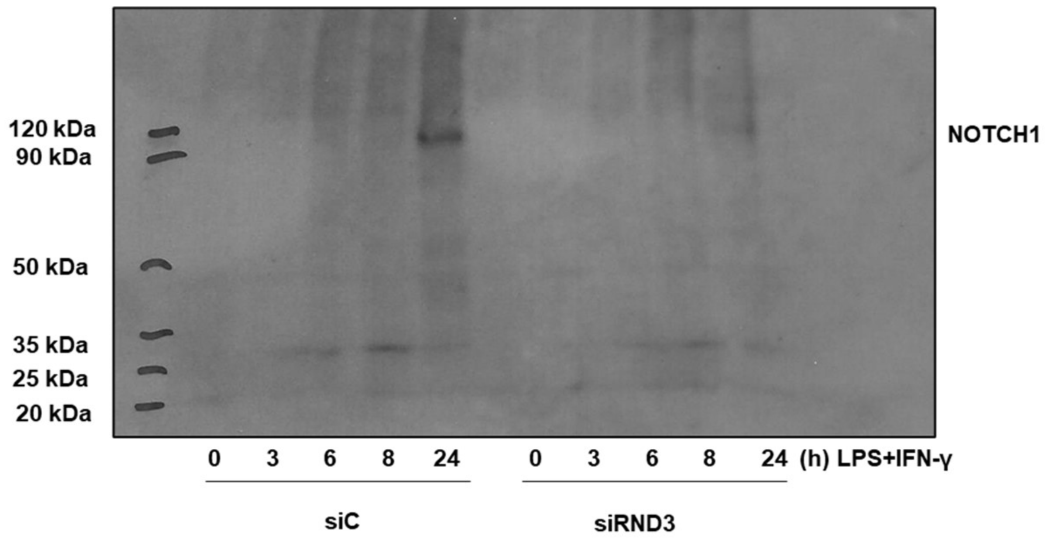
120 kDa
90 kDa
50 kDa
35 kDa
25 kDa
20 kDa
NOTCH1
0 3 6 8 24 0 3 6 8 24 (h) LPS+IFN-γ
siC
siRND3

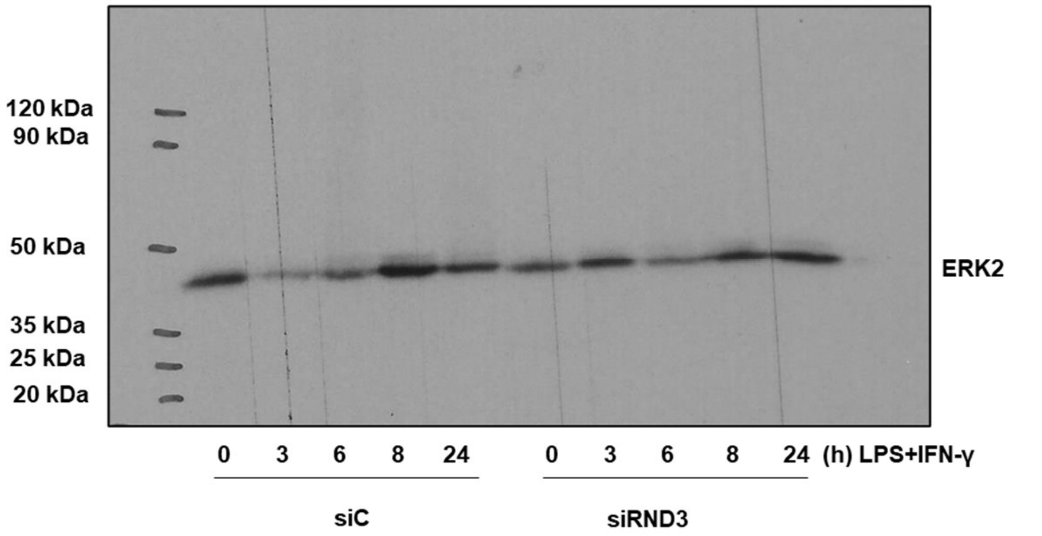
120 kDa
90 kDa
50 kDa
35 kDa
25 kDa
20 kDa
ERK2
0 3 6 8 24 0 3 6 8 24 (h) LPS+IFN-γ
siC
siRND3

Figure 3C

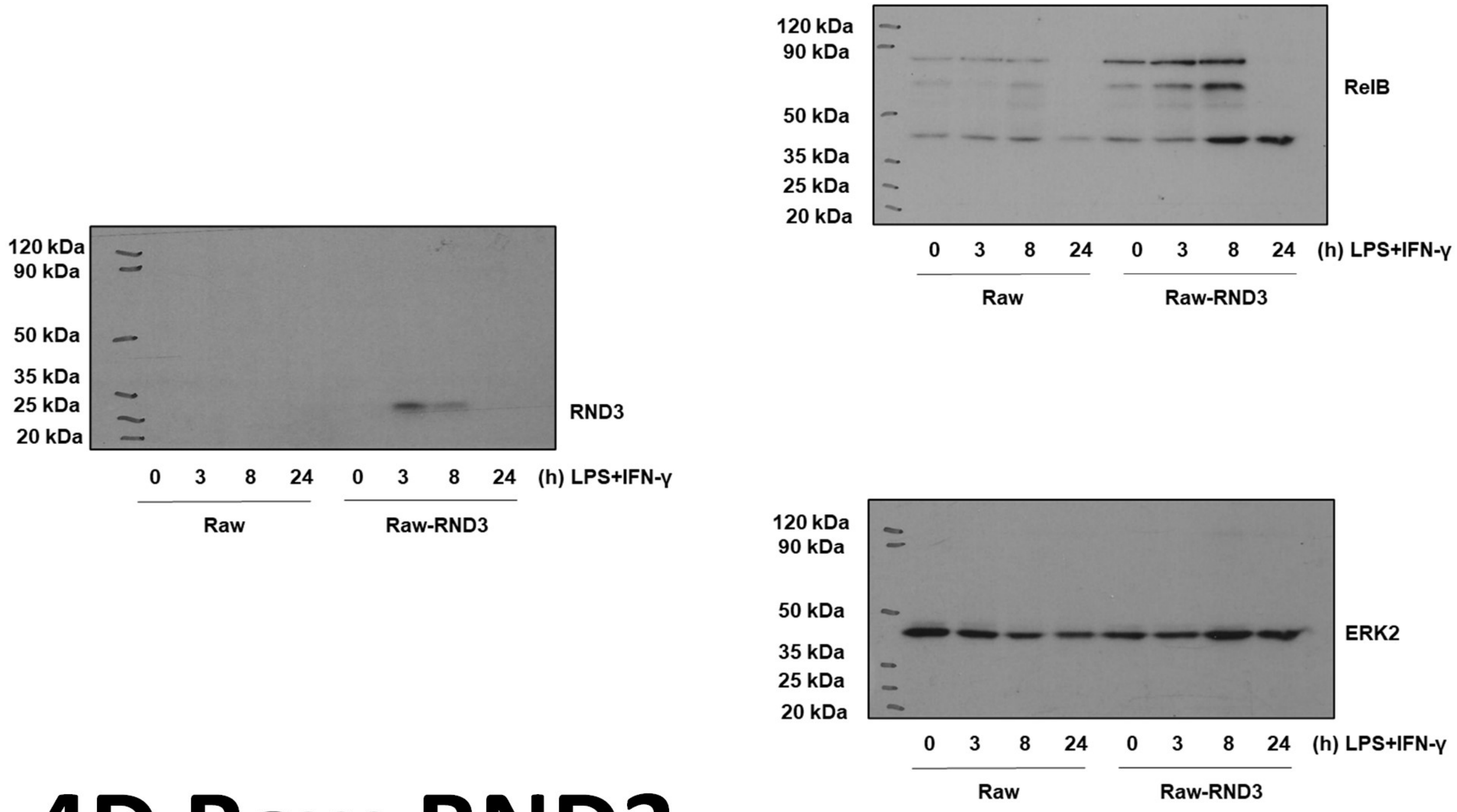

Figure 4D Raw-RND3

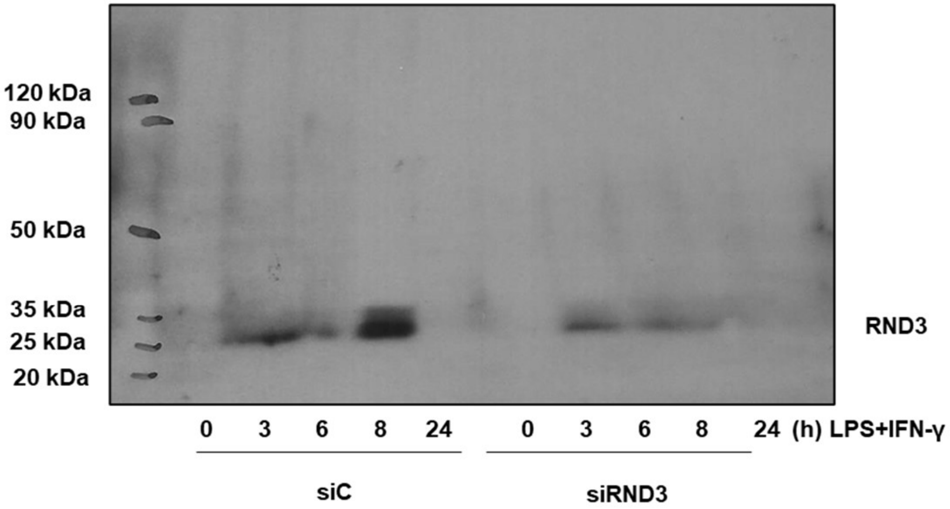

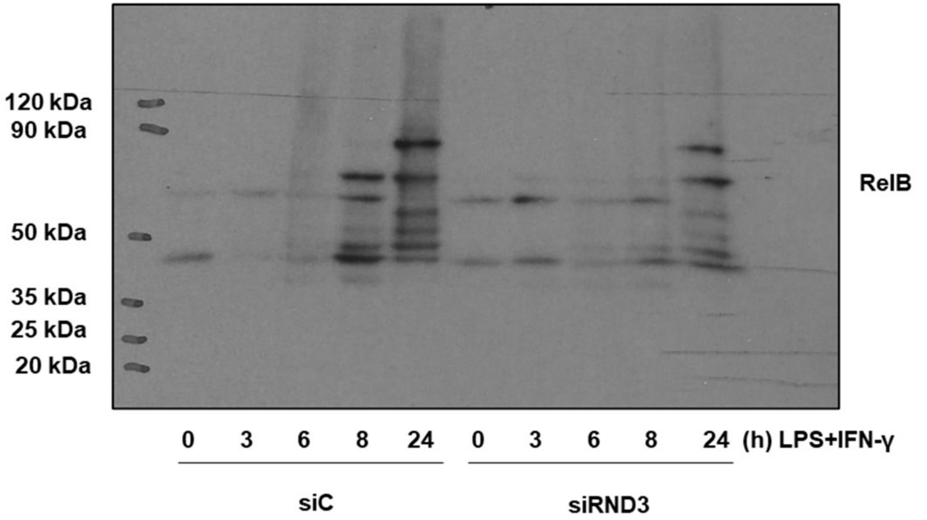

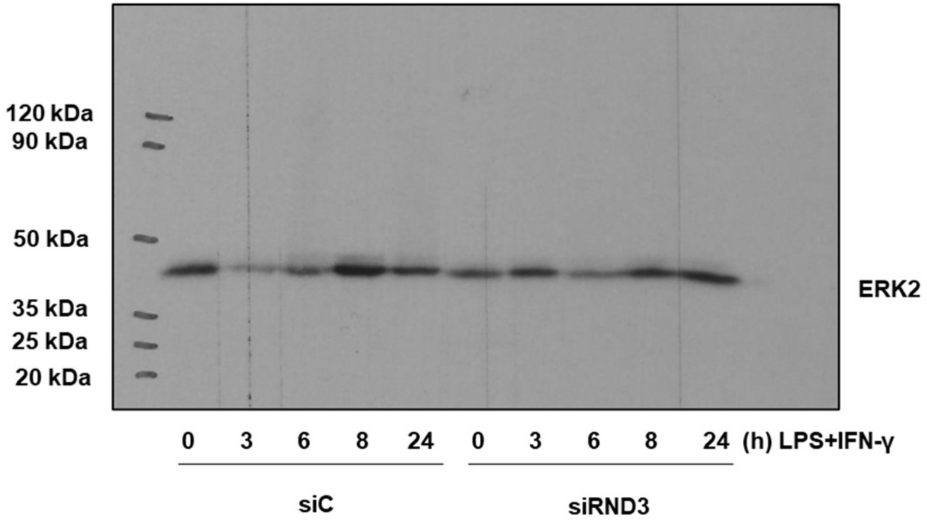

Figure 4D siRND3

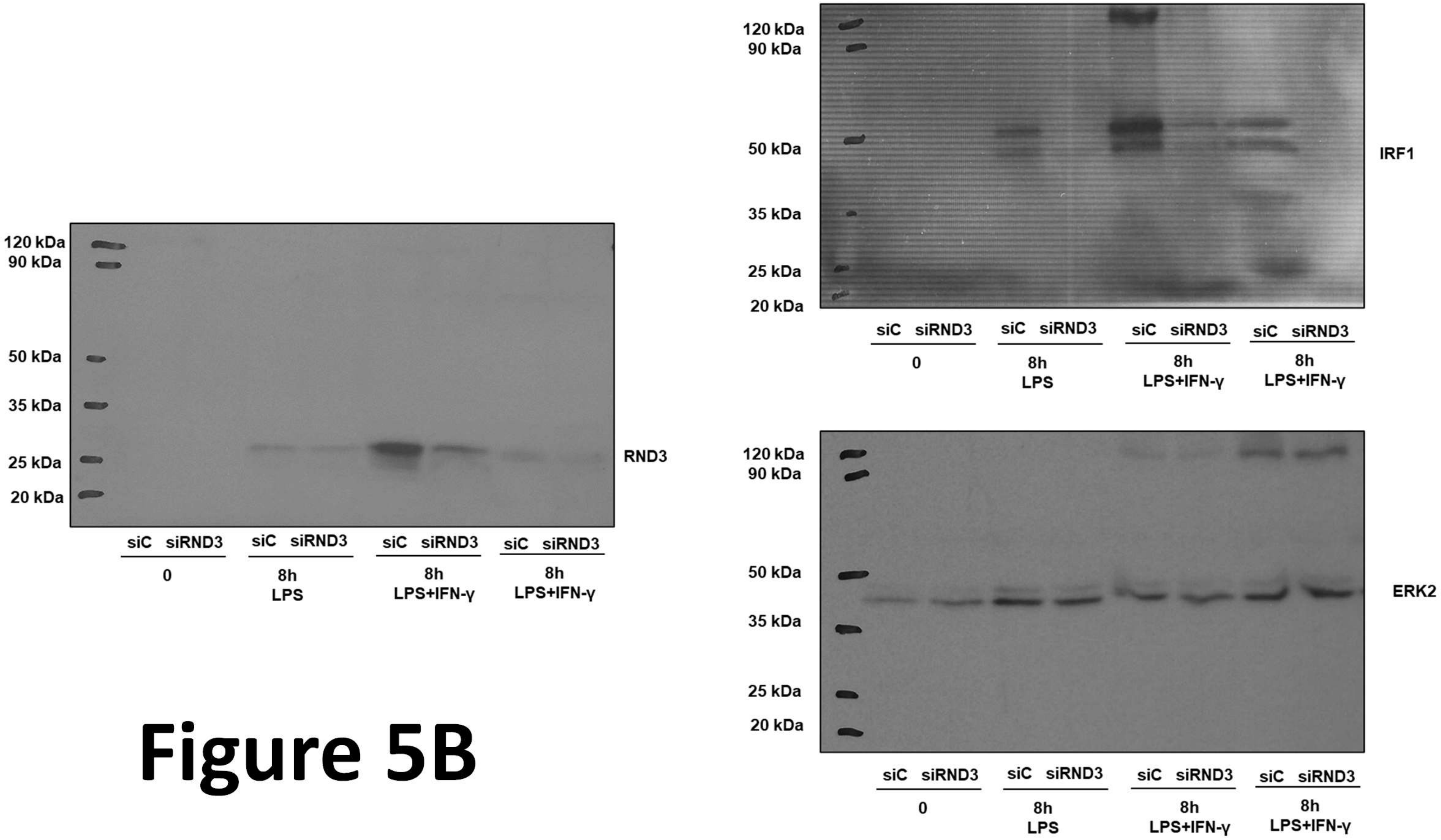
120 kDa
90 kDa
50 kDa
35 kDa
25 kDa
20 kDa
RND3
siC siRND3 siC siRND3 siC siRND3 siC siRND3
0
8h LPS
8h LPS+IFN-γ
8h LPS+IFN-γ
120 kDa
90 kDa
50 kDa
35 kDa
25 kDa
20 kDa
IRF1
siC siRND3 siC siRND3 siC siRND3 siC siRND3
0
8h LPS
8h LPS+IFN-γ
8h LPS+IFN-γ
120 kDa
90 kDa
50 kDa
35 kDa
25 kDa
20 kDa
ERK2
siC siRND3 siC siRND3 siC siRND3 siC siRND3
0
8h LPS
8h LPS+IFN-γ
8h LPS+IFN-γ

Figure 5B

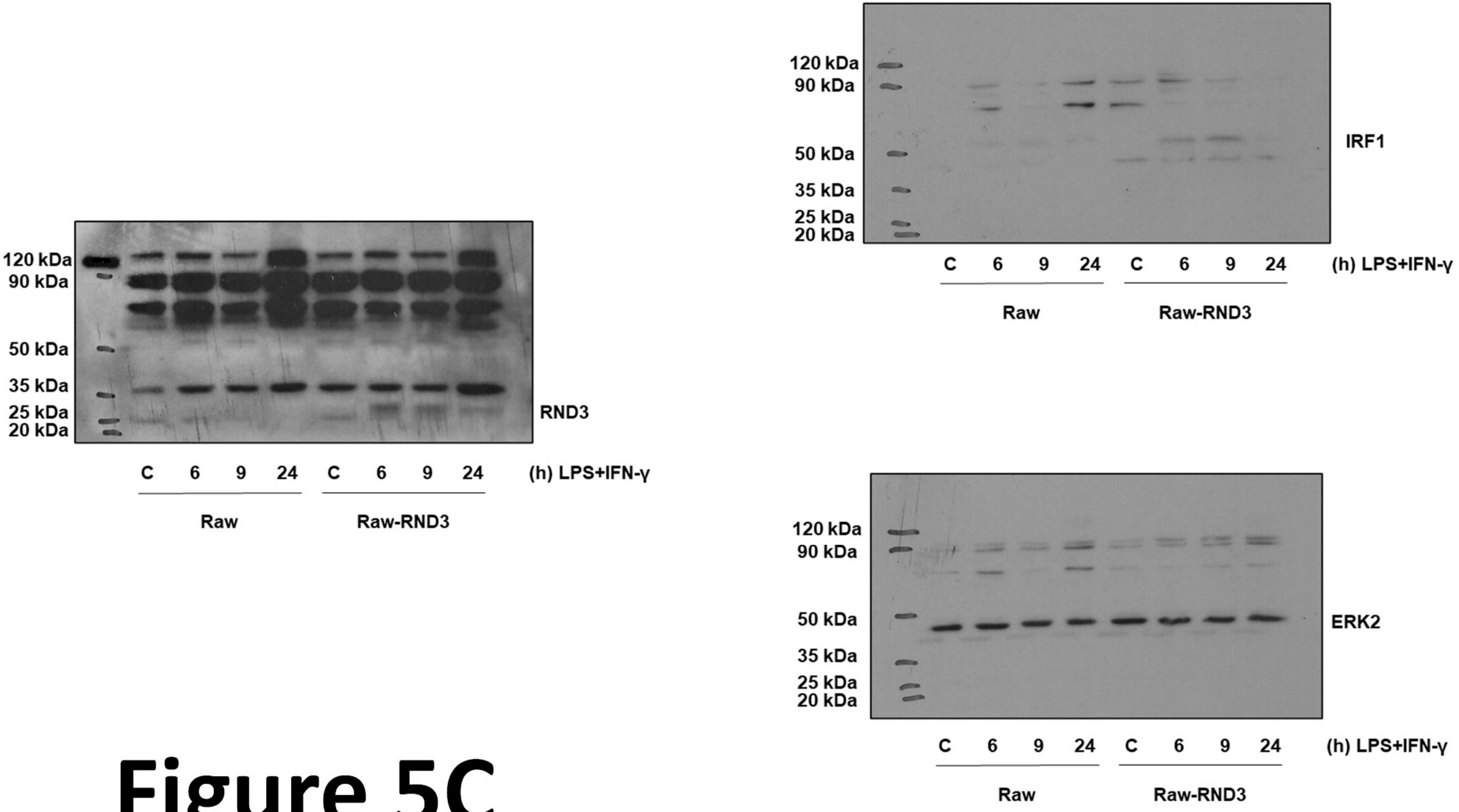
RND3
120 kDa
90 kDa
50 kDa
35 kDa
25 kDa
20 kDa
C 6 9 24 C 6 9 24 (h) LPS+IFN-γ
Raw
Raw-RND3
IRF1
ERK2

Figure 5C
